# Rethinking ethanol consumption and colorectal carcinogenesis: an insight from diet and gut microbiota

**DOI:** 10.3389/fcimb.2026.1761330

**Published:** 2026-06-02

**Authors:** Eva Gómez-Pérez, Sergio Ruiz-Saavedra, Aida Zapico, Adolfo Suárez, Ylenia Diaz, Carmen González del Rey, Nuria Salazar, Sonia González, Clara G. de los Reyes-Gavilán

**Affiliations:** 1 Department of Functional Biology, University of Oviedo, Oviedo, Spain; 2 Diet, Microbiota and Health Group, Instituto de Investigación Sanitaria del Principado de Asturias (ISPA), Oviedo, Spain; 3 Department of Microbiology and Biochemistry of Dairy Products, Instituto de Productos Lácteos de Asturias (IPLA-CSIC), Oviedo, Spain; 4 Digestive Service, Central University Hospital of Asturias (HUCA), Oviedo, Spain; 5 Digestive Service, Carmen and Severo Ochoa Hospital, Cangas del Narcea, Spain; 6 Department of Anatomical Pathology, Central University Hospital of Asturias (HUCA), Oviedo, Spain

**Keywords:** acetaldehyde, alcohol, colorectal cancer, conventional adenomas, dietary fibre, ethanol, intestinal polyps, microbiota

## Abstract

Colorectal cancer (CRC) is a pathology highly influenced by lifestyle and dietary habits, with ethanol consumption recognized as one of the most impactful and modifiable risk factors. Intestinal microbiota alterations have been observed both in high alcohol consumers and in the progression of intestinal polyps to CRC. The diet-microbiota-host interplay has a key role in health and disease. This perspective critically evaluates the mechanistic interplay within this triad and its potential role in mediating alcohol-induced toxicity, as well as its implications for the etiological link between alcohol consumption and CRC risk. Furthermore, we synthesize current advances and delineate key challenges for future research in this area. A particular focus is posed on the analysis of the anatomopathological, nutritional and microbiota data collected from individuals with intestinal polyps in a pilot study carried out in the region of Asturias, Northern Spain.

## 1 Introduction

Ethanol is responsible for about 5% of the global burden of disease and injury (WHO, 2020). It has been classified by the International Agency of Research on Cancer (IARC) within the Group 1 of carcinogens and was associated with 740.000 new cases of cancer each year globally (Rumgay et al., 2021). The relative risk of cancer appears to increase in a dose-dependent manner with alcohol consumption and may be further influenced by the concomitant exposure to other carcinogenic agents (Bagnardi et al., 2015). In light of this and other scientific evidences (GBD 2022a; GBD 2022b; Cherpitel et al., 2003; Manthey et al., 2019; Nyberg et al., 2022), the World Health Organization (WHO) firmly concluded that there is no safe level of ethanol consumption (WHO, 2020).

The WHO European Region accounts for the highest level of alcohol consumption in the world (WHO, 2020). Additionally to excessive alcohol intake, a shift in drinking behaviours with a marked rise in binge-drinking patterns has been noted (Carbia et al., 2023; Waszkiewicz et al., 2018). Spain had an average consumption per capita of pure alcohol of 10 litres in 2016, which increased to 12 litres in 2022, exceeding the mean intake estimated among the European Region (WHO, 2021; OECD/European Commission, 2025). The major body locations of cancers associated with ethanol consumption are, by increasing order of incidence, oropharynx, larynx, liver, oesophagus, oral cavity, colorectum and breast (Secretan et al., 2009). The high prevalence of CRC in the Western world may point to ethanol as an important dietary risk factor. Specifically, the GBD identified ethanol among the main CRC-related factors (GBD, 2022a), with intakes above 12 g/day increasing the risk of CRC by 8% (GBD, 2022b).

Asturias, located in northern Spain, ranks among the regions with the highest prevalence of alcohol consumption nationwide, occupying the second position for intake within the last 30 days and the third over the past 12 months (EDADES, 2024). Notably, higher CRC incidence and mortality rates have been observed in this region when compared with the national averages (AECC, 2018), suggesting a potential interplay between lifestyle factors and disease burden that warrants further investigation.

There is strong evidence of direct dose-response relationship between ethanol consumption and CRC (Cho et al., 2004). CRC has generally a slow development with the progression of precursor lesions on the intestinal mucosa taking long time to became malignant, which allows detection at early stages (intestinal polyps) and the application of strategies for primary prevention. Over 80% of CRC cases develop through the adenomatous pathway with typically characterized genetic alterations, originating conventional adenomas (CA) that harbour dysplastic cells. The serrated pathway is less frequent (about 20%) and includes hyperplastic polyps (harbouring hyperplastic cells) and other types of serrated polyps/lesions. Hyperplastic polyps usually present low risk of becoming malignant, whereas the risk of CA is higher (Sanabria et al., 2012).

## 2 Mechanisms of ethanol toxicity. A focus on the large intestine

Acetaldehyde is the primary and most toxic compound resulting from alcohol metabolism in the body. Oxidative metabolism mainly involves three groups of enzymes: Alcohol dehydrogenase (ADH) that oxidizes ethanol to acetaldehyde in the cell cytosol, Aldehyde dehydrogenase (ALDH) that oxidizes acetaldehyde to acetate in the mitochondria, and Cytochrome P450 2E1 (CYP2E1) that takes part of the ethanol oxidizing system to acetaldehyde in hepatic microsomes after acute or chronic alcohol consumption (Seitz and Becker, 2007). For several of these enzymes more than one genetic variant exists differing in their activity, which can result in either further acetaldehyde accumulation or faster conversion. Polymorphisms of ADH and ALDH play an important role in determining blood acetaldehyde levels among alcohol consumers (Quintanilla et al., 2005). The accumulation of acetaldehyde as related to the presence of specific alleles of these enzymes exhibit increased cancer risk (Yokoyama et al., 1998).

Highly reactive oxygen species (ROS) are generated during conversion of alcohol to acetaldehyde by CYP2E1. Acetaldehyde and ROS can interact with DNA building blocks to form DNA adducts. Acetaldehyde, ROS and DNA-adducts are cancer inducers (Seitz and Becker, 2007; Zakhari, 2006). A minimal non-oxidative metabolism of alcohol can also occur by two main pathways. One leads to the formation of fatty acid ethyl esters in a reaction catalysed by a fatty acid ethyl ester synthase. The other pathway leads to the formation of phosphatidyl ethanol in a reaction catalysed by the phospholipase D, thus altering the normal function of this enzyme and resulting in the production of phosphatidic acid (PA) from phosphatidylcholine. PA dysregulates the Mechanistic Target of Rapamycin pathway (mTOR), leading to nuclear translocation of β-catenin (Wnt signalling pathway) and transcription factor zonula occludens 1 (ZO-1)-associated nucleic acid binding (ZONAB) proteins with a consequent dysregulation of genes involved in cell proliferation and differentiation signalling (Na and Lee, 2017; Lima et al., 2010). Oxidative and non-oxidative pathways are interrelated and the inhibition of the oxidative pathways, increases the non-oxidative transformation (Werner et al., 2002; Zakhari, 2006).

Alcohol is absorbed primarily in the small intestine and transported to the liver, which is the main organ metabolizing this compound. However, other parts of the body also contribute to the metabolism of ethanol. Notably, alcohol concentration in the lumen of human large intestine is similar to that of the blood (Koehler et al., 2016). Cells from the intestinal mucosa along with the intestinal microbiota contribute to the metabolism of ethanol. The so-called “bacteriocolonic pathway of ethanol oxidation” can result in levels of acetaldehyde production that potentially exceeded the minimum mutagenic concentration (MMC) of this compound (estimated at 50-150 µM) (Salaspuro, 1997) and has been proposed to play an important role in ethanol-related CRC (Jokelainen et al., 1996; Salaspuro et al., 1999; Visapaa et al., 2002). CRC develops from initial lesions occurring in the intestinal mucosa, where there is higher oxygen content than in the lumen because of the diffusion of oxygen from the submucosa (Jokelainen et al., 1996). The production of acetaldehyde by microorganisms inhabiting the colorectal mucosa can contribute to increased ROS levels and the development of mucosa lesions (Jokelainen et al., 1996; Tsuruya et al., 2016a).

In addition, chronic alcohol intake causes intestinal mucosa inflammation, impairing mucosal integrity and epithelial surface erosions (Bishehsari et al., 2017; Na and Lee, 2017), whereas the production of proinflammatory cytokines and chemokines increases oxidative stress and activates the NF-κB nuclear factor, blocking apoptosis (Wang et al., 2015). Finally, high acetaldehyde concentrations can break down folic acid, an important vitamin in cell regeneration, thus enhancing mucosal damage (Giovannucci et al., 1995). All these factors together provide an altered mucosa microenvironment that facilitates tumour growth (**Supplementary Figure 1**).

## 3 The bacteriocolonic metabolism of ethanol and dietary fibres

The contribution to ethanol oxidation by different members of the intestinal microbiota and the mechanisms through which the microbiota modulates acetaldehyde levels in the colon is still poorly understood. Tsuruya et al. (2016a) determined *in vitro* the ability to accumulate acetaldehyde from ethanol at the MMC by faecal microbial isolates from Japanese alcoholics. The genus *Ruminococcus*, highly prevalent in the human gut microbiota and present in all donors, displayed the highest proportion of potential acetaldehyde accumulator members (PAAs) both in aerobic and anaerobic conditions; other less prevalent anaerobes such as *Prevotella, Collinsella, Coriobacterium* and *Bifidobacterium* also showed considerable abundance of PAAs. Interestingly, the presence of ROS in the culture medium enhanced acetaldehyde production by *Ruminococcus* and *Collinsella* PAAs, suggesting their potential ability to produce acetaldehyde under the aerobic conditions at the surface of the colorectal mucosa (Tsuruya et al., 2016a). Additionally, bacterial catalases of aerobic and facultative anaerobes also have the capacity to oxidize ethanol, contributing to acetaldehyde formation in the proximity of the intestinal mucosa (Tillonen et al., 1998) (**Supplementary Figure 1**).

Members of *Ruminococcus, Prevotella, Bifidobacterium*, and *Collinsella* genera are carbohydrates and dietary fibre fermenters in the gut (Liu et al., 2025; Precup and Voznar, 2019; Valentino et al., 2024) and all of them, together with the family *Coriobacteriaceae* (Zhao et al., 2022) have been positively associated with the intestinal production of short chain fatty acids, molecules with known health-related properties (Rios-Covian et al., 2016). *Ruminococcus* is a polyphyletic genus that includes two distinct clades assigned to families *Lachnospiraceae* and *Oscillospiraceae* (Abell et al., 2008; La Reau et al., 2016). Interestingly, species from *Lachnospiraceae* family encode for carbohydrate enzymes potentially involved in the metabolism of human N- and O-glycans (among them, *Ruminococcus torques* and *Ruminococcus gnavus*) whereas those from *Oscillospiraceae* seem to be more adapted to dietary fibre metabolism (Valentino et al., 2024). Notably, the representation of genomes from the *Oscillospiraceae* family was considerably higher in faecal samples from subjects with non-Westernized dietary patterns whereas *R. torques* was over-represented in Westernized subjects. These authors also evidenced a significant link between the *Lachnospiraceae* family and CRC. Although it is not currently known whether the same microorganism from a given genus or group (clade) could behave both as a fibre fermenter and as a PAA, or whether these abilities differ among microbial isolates, we could speculate that both types of microbiota metabolic activities (acetaldehyde conversion/accumulation and carbohydrate/fibre fermentation) are shaped by prolonged exposure to ethanol and/or dietary fibre, thus modulating the capacity of the microbiota to metabolize them and influencing long-term CRC risk.

## 4 The interplay of microbiota and ethanol in colorectal carcinogenesis

A depletion of microbial fibre fermenters/SCFA producers (*Bacteroides, Ruminococcus, Collinsella, Prevotella, Faecalibacterium, Roseburia*) together with an increase of facultative anaerobes, such as *Streptococcus* and members of the *Enterobacteriaceae* family has been repeatedly reported in faecal and intestinal mucosa samples of high ethanol consumers (Dubinkina et al., 2017; Gurwara et al., 2020; Jiao et al., 2022; Sosnowski and Przybylkowski, 2024), which reflect a potential alteration of the gut fibre and carbohydrate metabolism in these individuals. These shifts could be partly explained by the susceptibility of obligate anaerobes to ROS and oxygen, which may become elevated in the intestinal environment, mainly at the mucosal level, as a result of chronic ethanol exposure (Tsuruya et al., 2016b). Relating to that, Tsuruya et al. hypothesized that the apparently contradictory lower conversion of ethanol to acetaldehyde found by them in faecal cultures of alcohol consumers as compared to non-alcohol consumer volunteers may be explained by a shift in the conversion of ethanol to acetaldehyde from the lumen towards the intestinal mucosa due to the increased concentration of oxygen and ROS at this location (Tsuruya et al., 2016b); however, this hypothesis must be corroborated. On the other hand, different trends of microbiota alterations have been reported in intestinal adenomas and CRC, which varied depending on the severity of intestinal mucosal lesions, ethnicity, dietary habits, and other factors. These alterations globally consist of one or more of these patterns: a) an increase in specific pathogens, including oral pathogens (*Fusobacterium nucleatum, Parvimonas micra, Escherichia coli, Bacteroides fragilis, Shigella flexneri, Campylobacter ureolyticus, Selenomonas sputigena, Dialister pneumosintes*, among others), b) alterations in the levels of methanogenic archaea (*Methanobrevibacter*), c) the increase of facultative anaerobes (*Streptococcus*, Proteobacteria and specifically some members of the family *Enterobacteriaceae*) and d) the depletion of fibre fermenters and SCFA producers (*Ruminococcus, Collinsella, Prevotella, Bacteroides*, among others) (Clavenna et al., 2023; Elkholy et al., 2023; Flemer et al., 2017, Flemer et al., 2018; Heng et al., 2023; Leung et al., 2019; Li et al., 2022; Lu et al., 2016; Senthakumaran et al., 2023).

Analysing tumour and adjacent normal mucosa from patients at different stages of CRC Mouradov et al. identified three oncomicrobial community subtypes that differed in tumour features and prognosis. Notably, one of these subtypes (representing 44% of tumours) was limited to left-sided tumours, stages II-III and chromosome instability as genetic signature and presented as the main microbial differential characteristic a depletion of multiple core gut commensals of Firmicutes (Bacillota)/Bacteroidetes (Bacteroidota) phyla related with carbohydrate fermentation but still retaining potential for colonic saccharolytic fermentation. This oncomicrobial subtype displayed higher survival than the other two groups whose altered microbiota composition presented switches to proteolytic and fatty acids metabolism, respectively as alternative energy sources, with potential pathobiological consequences (Mouradov et al., 2023).

Interestingly, gut microbiota alteration trends reported for high alcohol consumers share some common features with the microbiota of individuals with CA and CRC, such as a higher presence of facultative anaerobes and lower of fibre fermenters, as well as with the oncomicrobial community subtype of Mouradov et al. with better prognosis (Mouradov et al., 2023). Further illustrating the relationship between colonic fermentation and ethanol consumption, a recent study in rats demonstrated that the intake of some fermentable dietary fibres (inulin, pectin, and guar gum) reduced voluntary alcohol intake and modulated the gut microbiota, enhancing resilience after alcohol intoxication (Calleja-Conde et al., 2025).

Currently, it is not deeply known the dynamics of acetaldehyde production/conversion in the large intestine of alcohol consumers, nor the dynamics of ethanol-induced microbiota alterations in CRC development. Additionally, the specific microorganisms involved in this process have yet to be fully identified. It is neither known how and to what extent alterations of the intestinal microbiota profiles, the depletion on “beneficial” fibre fermenter/SCFA producer microorganisms and the altered levels of facultative anaerobes could affect/modify the microbial metabolism of ethanol at the intestinal level and to what extent it could influence the onset, progression and prognosis of ethanol-related mechanisms of CRC.

## 5 Precancerous intestinal lesions and intestinal microbiota. How far is consumption of ethanol and dietary fibre important?

In a previous study conducted by our research team, we compared dietary habits of a reduced group of adults diagnosed with intestinal polyps and of nonpathological controls undergoing colonoscopy and histopathological analyses of the resected intestinal mucosa at two Hospitals in Asturias Region, Northern Spain. Among the dietary potential carcinogens studied, ethanol and dibenzo (a) anthracene were consumed significantly more by individuals with intestinal polyps, both linked to the consumption of alcoholic beverages, whereas the intake of wholegrain cereals was associated to a reduced risk of belonging to the polyp’s group (Ruiz-Saavedra et al., 2022). As part of the same project (Effect of diet and exposure to xenobiotics generated during food processing on the genotoxic/cytotoxic capacity of the intestinal microbiota-MIXED), we studied the faecal microbiota composition from 54 adults by 16S rRNA gene-based metataxonomic analyses, to determine the microbiota alterations as related with the degree of intestinal mucosal lesions. Deeper microbiota alterations were found in individuals with CA than in those with hyperplastic polyps. *Ruminococcus torques* was enriched in individuals with both types of polyps. In individuals with CA, decreased relative abundances of some carbohydrate fermentative families (*Christensenellaceae*, *Erysipelotrichaceae* and *Prevotellaceae*), of the facultative anaerobe and fermentative family *Streptococcaceae* and of the metanogenic *Methanobacteriaceae*, were the main signatures found linked to the degree of dysplasia, reflecting the progressive alteration of gut microbiota populations involved in fermentation processes in accordance with a greater severity of the intestinal mucosa lesions (Ruiz-Saavedra et al., 2024).

Considering the results commented just above, we examined in more detail the alcohol and fibre consumption and the associated polyp’s risk of those individuals participating in our previous study (Ruiz-Saavedra et al., 2024). Dietary questionnaires were collected for 41 of these volunteers, using the methodology and statistical analyses previously described (Ruiz-Saavedra et al., 2022). Individuals with CA had significantly higher consumption of ethanol as compared to the control group, independently of the grade of dysplasia (**Table 1**), mainly related with the comparatively higher intake of cider (a regional drink elaborated by fermentation of apple juice), especially among those subjects presenting high grade dysplasia. Individuals with low grade dysplastic adenomas consumed less total and soluble fibre in comparison with controls (**Table 1**). A Firth penalized logistic regression model adjusted by age, gender and body mass index, evidenced a significant strong association between ethanol intake and the presence of CA (Odds ratio 7.5) when doses exceed 7.90 g/day (**Table 2**). Among alcoholic beverages, the consumption of more than 66 ml/day of beer presented an increased risk tendency of belonging to the CA group (Odds ratio 7.30). In contrast a greater intake of dietary soluble fibre consumption (mostly derived from vegetable origin food sources-data not shown) resulted protective towards CA. Indeed, consumption of soluble fibre over 2.85 g/day showed a protective effect, being negatively associated with CA risk (Odds ratio 0.14) (**Table 2**). No significant associations were found between the consumption of ethanol and the risk of presenting hyperplastic polyps.

**Table 1:** Intake of ethanol and its dietary sources and of total, soluble and insoluble dietary fibre as related to histopathological lesions of the intestinal mucosa.

Intake of ethanol and its dietary sources and of dietary fibre	Controls (N = 14)	Hyperplastic polyps (N = 7)	Conventional adenomas (N = 20)	
			LGD (N = 16)	HGD (N = 4)
Ethanol (g/day)	4.00 ± 6.26	17.20 ± 32.63	14.29 ± 18.86 *	
			13.42 ± 18.96 *	17.80 ± 20.88 *
Main dietary sources:				
Beer (ml/day)	50.50 ± 92.24	37.02 ± 55.35	99.95 ± 148.03	
			103.62 ± 162.45	85.25 ± 80.52
Red wine (ml/day)	1.07 ± 2.89	157.14 ± 373.53	53.82 ± 170.36	
			67.27 ± 189.20	0.00 ± 0.00
Cider (ml/day)	15.60 ± 40.35	6.67 ± 17.64	138.38 ± 337.27 *	
			67.03 ± 118.11	423.75 ± 717.58 *
White wine (ml/day)	12.86 ± 26.37	6.19 ± 14.96	12.25 ± 16.01	
			10.62 ± 15.79	18.75 ± 17.50
Total fibre (g/day)	23.62 ± 9.01	19.42 ± 5.49	19.65 ± 6.37	
			17.94 ± 5.28*	26.52 ± 6.23
Insoluble fibre (g/day)	13.78 ± 5.86	12.40 ± 3.71	11.61 ± 4.92	
			10.30 ± 3.40	16.87 ± 7.04
Soluble fibre (g/day)	2.62 ± 0.99	2.39 ± 0.63	2.27 ± 0.96	
			2.06 ± 0.79*	3.11 ± 1.24

Data are presented as mean ± standard deviation. *Statistically significant differences (p < 0.05) of the group of hyperplastic polyps or conventional adenomas compared to the control group by Mann-Whitney U test. LGD, low grade dysplasia. HGD, high grade dysplasia. N, number of individuals within each group.

**Table 2:** Firth’s penalized logistic regression analysis of the risk of being diagnosed with conventional adenomas associated to the intake of ethanol and its dietary sources and to the intake of dietary fibre.

Intake of ethanol and its dietary sources and of dietary fibre	N (% conventional adenomas)	Mean ± SD	OR (95% CI)	p-value
Ethanol (g/day)				
≤ 2.21	13 (30.77)	0.77 ± 0.99	0.00 (0.00-2.94)	0.091
> 2.21 to ≤ 7.90	10 (70.00)	4.80 ± 2.16	3.24 (0.59-20.62)	0.177
> 7.90	11 (81.82)	25.80 ± 19.92	7.50 (1.26-70.64)	**0.026**
Main dietary sources:				
Beer (ml/day)				
≤ 0.00	13 (38.46)	0.00 ± 0.00	0.00 (0.00-0.28)	**0.039**
> 0.00 to ≤ 66.00	12 (66.67)	52.06 ± 20.90	0.81 (0.23-2.56)	0.148
> 66.00	9 (77.78)	231.26 ± 174.58	7.30 (1.00-86.93)	**0.050**
Red wine (ml/day)				
≤ 0.00	27 (55.56)	0.00 ± 0.00	0.00 (0.00-5.38)	0.136
> 0.00	7 (71.43)	155.90 ± 270.60	1.94 (0.39-12.63)	0.429
Cider (ml/day)				
≤ 0.00	19 (42.11)	0.00 ± 0.00	0.00 (0.00-7.89)	0.158
> 0.00 to ≤ 50.00	5 (80.00)	27.17 ± 22.42	4.15 (0.58-49.58)	0.161
> 50.00	10 (80.00)	285.00 ± 441.06	3.33 (0.59-23.93)	0.176
White wine (ml/day)				
≤ 0.00	18 (50.00)	0.00 ± 0.00	0.01 (0.00-8.59)	0.173
> 0.00 to ≤ 10.00	5 (60.00)	8.67 ± 1.83	1.09 (0.15-8.68)	0.933
> 10.00	11 (72.73)	34.70 ± 23.65	1.83 (0.39-9.59)	0.444
Total fibre (g/day)				
≤ 17.43	12 (66.67)	13.80 ± 1.94	0.03 (0.00-23.71)	0.317
> 17.43 to ≤ 23.74	11 (63.64)	20.89 ± 2.05	0.69 (0.12-3.97)	0.677
> 23.74	11 (45.45)	29.85 ± 6.25	0.33 (0.05-1.83)	0.207
Insoluble fibre (ml/day)				
≤ 9.32	12 (66.67)	7.41 ± 1.26	0.18 (0.00-196.27)	0.637
> 9.32 to ≤ 13.45	11 (72.73)	11.89 ± 1.44	1.07 (0.18-6.61)	0.942
> 13.45	11 (36.36)	18.68 ± 4.13	0.30 (0.05-1.72)	0.180
Soluble fibre (g/day)				
≤ 1.87	11 (72.73)	1.40 ± 0.30	0.12 (0.00-161.33)	0.571
>1.87 to ≤ 2.85	12 (75.00)	2.30 ± 0.31	0.94 (0.15-6.05)	0.950
> 2.85	11 (27.27)	3.55 ± 0.60	0.14 (0.02-0.84)	**0.031**

The model has been adjusted by age, gender and body mass index. The low tertile of the control group was used as reference for the analysis. CI, confidence interval; OR, Odds ratio; SD, standard deviation; N, number of individuals within each consumption range.

Bold numbers indicate statistical significance at p-value < 0.05.

## 6 Discussion and future perspective

The wide confidence interval for ethanol and fibre consumption and the low number of individuals exposed to the target parameters reduces the precision and limits the strength of our analysis. Despite this, our findings suggest a strong influence of ethanol exposure in the studied population group and points to its consumption as one of the main risk factors for the presence of CA. Although no relationship was found for ethanol or fibre with the presence of hyperplastic polyps, the differential higher abundance of *R. torques* in individuals with different types of intestinal polyps may be linked with more westernized dietary habits that may predispose to a higher consumption of ethanol and lower of fibre. Although wide epidemiological surveys and further longitudinal dietary intervention studies are warranted to establish the link among diet, alcohol consumption and microbiota, these preliminary results contribute to support the usefulness of dietary fibre consumption and reduction of alcohol intake in the prevention of the onset and progression of precancerous intestinal mucosal lesions (**Supplementary Figure 1**).

It is of high interest to expand our knowledge on how the progressive alterations of the intestinal microbial populations involved in fermentative processes could influence the colonic fermentation and the metabolism of ethanol, fibre and other dietary components and how the different consumption patterns of ethanol could affect these processes. Identifying biomarkers of early microbiota alteration in relation to ethanol-induced gut mucosa lesions may strengthen primary CRC prevention.

Finally, it is advisable to implement public health policies aimed at limiting alcohol consumption and raising public awareness of CRC risks associated with ethanol, especially in populations where the consumption of alcohol is associated with social and cultural habits.

## Data Availability

The original contributions presented in the study are included in the article. Further inquiries can be directed to the corresponding author.
