# Supplementary figures and images for: Rethinking ethanol consumption and colorectal carcinogenesis: an insight from diet and gut microbiota
Source: Front Cell Infect Microbiol. 2026 Jun 2;16:1761330. doi: 10.3389/fcimb.2026.1761330 (PMC13269019; doi:10.3389/fcimb.2026.1761330)

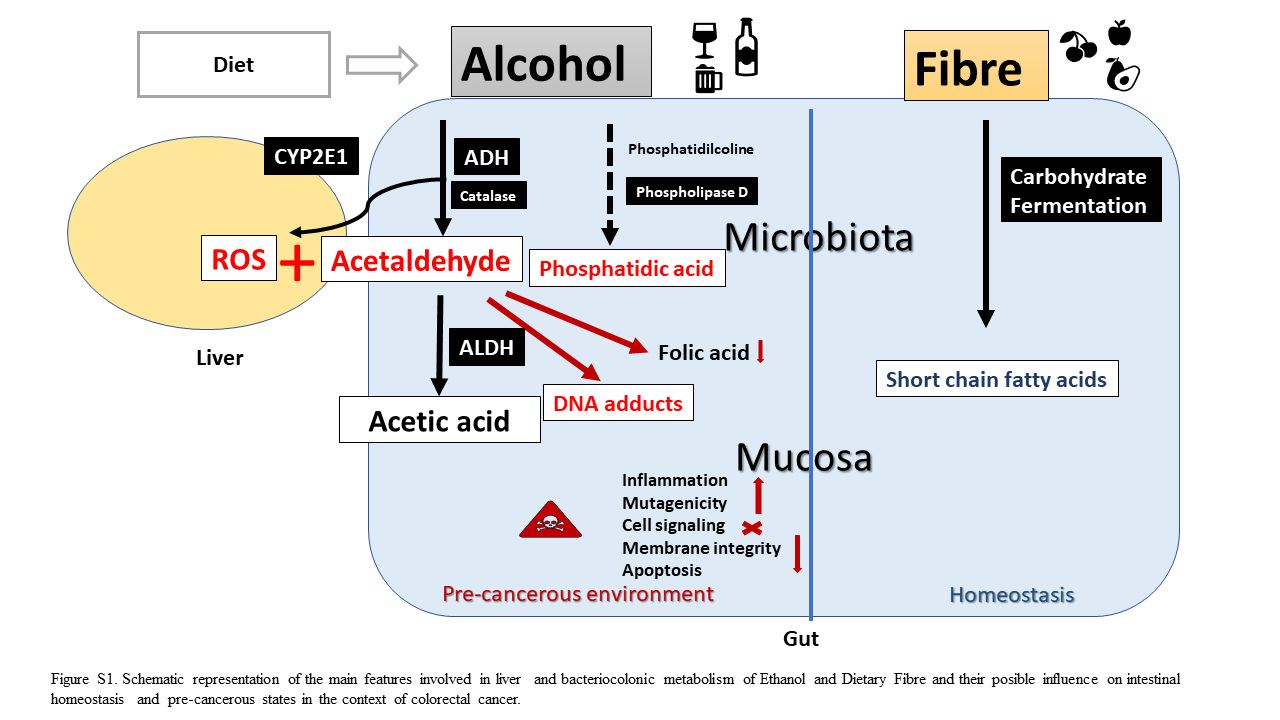

Supplement: Supplementary file 1 [file Image1.png]
